# Quality Improvement Intervention Lowers Hypothermia Rates in Preterm Infants in a Resource‐Limited Setting

**DOI:** 10.1111/ped.70397

**Published:** 2026-04-15

**Authors:** Patrycia Maria Gomes da Fonte Rosa, Juliana Dantas de Araújo Santos Camargo, Sávio Ferreira Camargo, Amaxsell Thiago Barros de Souza, Camiliane Azevedo Ferreira, Amanda Karoline da Costa Bezerra, Raionara Cristina de Araújo Santos, Cijara Leonice de Freitas, Ricardo Ney Cobucci, Fabiana Ariston Filgueira, Anna Christina do Nascimento Granjeiro Barreto

**Affiliations:** ^1^ Maternity Hospital‐School Januário Cicco (MEJC) Natal Rio Grande do Norte Brazil; ^2^ Federal University of Rio Grande Do Norte (UFRN) Natal Rio Grande do Norte Brazil; ^3^ Brazilian Company of Hospital Services (EBSERH) Natal Rio Grande do Norte Brazil

**Keywords:** neonatal hypothermia, neonatal intensive care unit, preterm infants, quality improvement, resource‐limited settings

## Abstract

**Background:**

Admission hypothermia significantly threatens preterm neonates, especially in resource‐limited settings.

**Methods:**

A quality improvement (QI) initiative focusing on thermoregulation was implemented in a Brazilian NICU. We conducted a quasi‐experimental pre‐post study comparing preterm infants (< 1500 g or < 33 weeks gestation) admitted before (*n* = 63) and after (*n* = 88) the intervention. Primary outcome was hypothermia prevalence (< 36.5°C) within the first hour of life.

**Results:**

The QI intervention reduced hypothermia prevalence from 82.5% to 68.2% (crude PR = 0.83; 95% CI: 0.69–0.99; *p* = 0.047) and increased median admission temperature from 35.3°C to 35.8°C (*p* = 0.013). Post‐intervention hypothermia was associated with increased mortality (PR = 3.27; 95% CI: 1.06–10.05; *p* = 0.017).

**Discussion:**

Implementing targeted QI measures improved thermal outcomes among preterm neonates in a resource‐constrained NICU. Despite reductions, persistent hypothermia highlights the need for ongoing efforts to enhance neonatal care and survival.

## 1 Introduction

Neonatal hypothermia, defined as an axillary temperature below 36.5°C, remains a critical challenge in neonatal care. Severity is classified as mild (36.0°C–36.4°C), moderate (32.0°C–35.9°C), or severe (< 32.0°C). Preterm neonates, especially those with very low birth weight (< 1500 g) or gestational age under 33 weeks, are particularly vulnerable due to immature thermoregulatory mechanisms and a high surface area‐to‐volume ratio, which together increase their risk of rapid heat loss through conduction, convection, evaporation, and radiation [1].

Admission hypothermia upon entry to the neonatal intensive care unit (NICU) is strongly correlated with adverse outcomes, including increased risks of pulmonary hemorrhage, sepsis, intraventricular hemorrhage, and overall mortality among this fragile population [2, 3]. The global impact of neonatal hypothermia remains substantial, particularly in resource‐constrained environments.

For instance, a cross‐sectional study conducted in Ethiopia documented a hypothermia prevalence of 61.5% among neonates within the first 6 h after birth, identifying low birth weight and the absence of skin‐to‐skin contact as significant contributing factors [4]. Similarly, research in Rwanda revealed a 54.8% prevalence of hypothermia at NICU admission, associated with prematurity, cesarean delivery, and the need for resuscitation; notably, hypothermic infants in this cohort faced an 18.9% mortality rate [3].

A study from 2017 to 2018 at a tertiary referral center in Natal, Brazil, reported alarmingly low rates of normothermia (3.9%) among very low birth weight preterm infants upon NICU admission. The majority (85.9%) presented with moderate hypothermia (mean temperature: 34.7°C), which correlated with poorer neonatal outcomes [5]. These findings underscore the urgent need for effective thermal care strategies.

Recognizing the detrimental effects of hypothermia, quality improvement (QI) initiatives have emerged as promising strategies to enhance thermal care. Such interventions often involve the implementation of standardized protocols, targeted staff education, and the reinforcement of evidence‐based practices [6]. In 2022, professionals from a maternity hospital in northeastern Brazil in partnership with doctors from a French maternity hospital instituted a Standard Operating Procedure (SOP) specifically designed to improve neonatal thermoregulation. This initiative included comprehensive staff training delivered through workshops and simulation exercises aimed at optimizing thermal management practices within the delivery room environment.

Therefore, this study aimed to evaluate the effectiveness of the QI protocol implemented on the thermal outcomes of preterm neonates admitted to the NICU of the Januário Cicco Maternity School, Natal, Brazil. Specifically, the primary objective was to determine the impact of the intervention on the prevalence of hypothermia within the first hour of life among infants born weighing < 1500 g and/or at < 33 weeks gestation, comparing pre‐ and post‐intervention periods. Secondary objectives included assessing changes in median admission temperatures and examining the association between admission hypothermia and neonatal mortality.

## 2 Materials and Methods

### 2.1 Study Design and Setting

A quasi‐experimental pre‐post intervention study design was employed to evaluate the impact of a QI initiative. This design involved comparing neonatal outcomes during two distinct 6‐month periods: a pre‐intervention phase (July–December 2021) and a post‐intervention phase (January–June 2023). Data were collected retrospectively from medical records. The study was conducted within the NICU of the Januário Cicco Maternity School, Natal, Brazil. The NICU is designated as type II according to the Brazilian Ministry of Health, equipped with 20 beds, and experiences a preterm birth rate of approximately 28%–30%.

### 2.2 Quality Improvement Intervention

**FIGURE 1:**
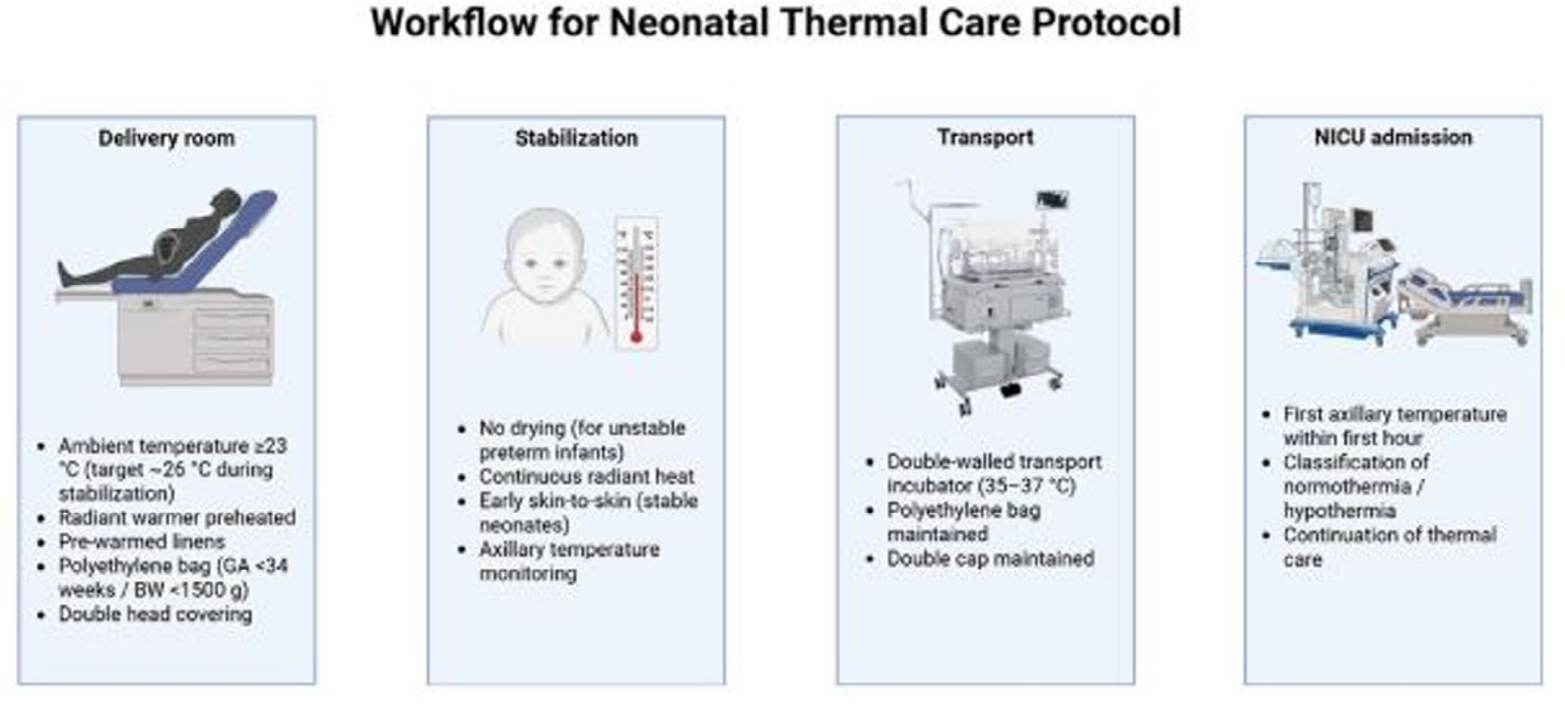
Neonatal thermal management workflow.

In response to previously documented high rates of neonatal hypothermia at the institution [5], a SOP for thermoregulation during delivery and subsequent admission to the NICU was developed in 2022 (Supporting Information S1) and formally implemented as a QI intervention in January 2023. The thermal management workflow is illustrated in Figure 1. The protocol was incorporated into routine clinical practice and applied to preterm and/or very low birth weight neonates admitted to the NICU during the post‐intervention period. Implementation activities were initiated prior to the post‐intervention phase to ensure full integration of the SOP into standard care workflows.

The intervention encompassed thermal care from birth through NICU admission. Delivery rooms and operating rooms were maintained at ambient temperatures above 23°C, with reinforcement of a target temperature of approximately 26°C during neonatal stabilization and resuscitation. Operationally, > 23°C was adopted as the institutional minimum standard, while the higher target was emphasized in high‐risk situations. Ambient temperatures were monitored using wall‐mounted thermometers routinely available in delivery and operating rooms. Prior to delivery, radiant heat sources were preheated for at least 30 min, and resuscitation surfaces, surgical fields, linens, neonatal caps, and transport incubators were systematically pre‐warmed.

Immediately after birth, eligible neonates (birth weight < 1500 g and/or gestational age < 33 weeks) received active thermal protection according to the SOP. Neonates were placed on pre‐warmed fields under a radiant heat source. Clinically unstable preterm neonates under 34 weeks of gestation and/or weighing < 1500 g were not dried and were placed in a polyethylene bag to reduce evaporative heat loss, which was maintained until thermal stabilization in the neonatal unit. Double head covering was recommended, and for extremely low birth weight infants (< 1000 g), the use of a chemical thermal mattress was recommended when available.

For clinically stable neonates, including selected preterm infants under 34 weeks, the protocol emphasized early skin‐to‐skin contact with the mother during the first hour after birth (Golden Hour), under continuous supervision by the healthcare team. Routine procedures, such as weighing, anthropometric measurements, and administration of prophylactic medications, were deferred until completion of this period. Breastfeeding initiation was encouraged as soon as the neonate demonstrated readiness, according to maternal preference and clinical condition.

Transport from the Obstetric Center or Obstetric Surgical Center to the NICU was performed in a double‐walled transport incubator for preterm infants under 34 weeks, with programmed temperatures ranging from 35°C to 37°C. Polyethylene bags and double head covering were maintained during transfer when applicable. Axillary temperature was routinely measured within the first hour of life upon NICU admission as part of standard clinical care.

Although the SOP includes stratified recommendations based on gestational age, birth weight thresholds, and neonatal clinical stability, the present study focused exclusively on neonates meeting the inclusion criteria of birth weight < 1500 g and/or gestational age < 33 weeks. All neonates in the post‐intervention group received thermoregulation measures appropriate to their gestational age, birth weight, and clinical condition, as defined in the SOP. Clinically unstable neonates were managed according to the same thermal protection principles, with deviations limited to immediate life‐saving interventions.

Implementation of the QI intervention was supported by a structured two‐phase educational program. The first phase consisted of sensitization workshops delivered via 20‐min expository lectures, reaching 60 staff members, including physicians, nurses, and nursing technicians from the Obstetric Center and the Obstetric Surgical Center. Knowledge acquisition in this phase was evaluated through written post‐tests. The second phase involved high‐fidelity clinical simulations using neonatal manikins, which included 18 staff members in 20‐min practical sessions. Adherence to the protocol was monitored through routine review of delivery room and NICU medical records, including documentation of thermal protection measures and axillary temperature measurements. Feedback to staff was provided during routine service discussions when deviations were identified.

### 2.3 Study Population and Eligibility Criteria

The study population comprised all preterm neonates admitted to the NICU with a birth weight < 1500 g and/or a gestational age < 33 weeks during the specified pre‐ and post‐intervention periods. Neonates were excluded if they were presented with congenital infections, major congenital malformations, or diagnosed inborn errors of metabolism. Additionally, infants transferred from other facilities more than 24 h after birth or those with missing axillary temperature data recorded within the first hour of life were excluded from the analysis.

### 2.4 Data Collection and Variables

Data were systematically extracted from patient medical records and hospital administrative registries. Key variables collected included: maternal characteristics (age, number of prenatal visits, presence of hypertensive disorders, gestational diabetes, urinary tract infections during pregnancy, administration of antenatal corticosteroids); delivery characteristics (mode of delivery, singleton versus multiple gestation); and neonatal characteristics—sex, gestational age at birth, birth weight, birth weight z‐score calculated using Intergrowth‐21st standards, classification as small for gestational age [SGA, defined as birth weight < 10th percentile for gestational age], requirement for delivery room resuscitation, Apgar scores at 1 and 5 min, use of continuous positive airway pressure [CPAP] in the delivery room, initial axillary temperature recorded within the first hour of life, and final hospital outcome (discharge or death). Neonatal mortality was further categorized based on timing: early neonatal (death occurring between 0 and 6 days of life), late neonatal (7–27 days), and post‐neonatal (≥ 28 days).

### 2.5 Statistical Analysis

The distribution of continuous variables was assessed for normality using the Kolmogorov–Smirnov test. Descriptive statistics were generated: continuous variables exhibiting non‐normal distributions were summarized using medians and interquartile ranges (IQR), while categorical variables were presented as frequencies and percentages (%). Bivariate comparisons between the pre‐ and post‐intervention groups were conducted using Chi‐square tests (or Fisher's exact test where appropriate) for categorical variables and Mann–Whitney U tests for continuous variables [7, 8].

Prevalence ratios (PR) and corresponding 95% confidence intervals (95% CI) were calculated directly from 2 × 2 contingency tables using the logarithmic (Wald) method. All PRs reported in this study are crude (unadjusted) estimates, as analyses were restricted to bivariate comparisons between study period and outcomes of interest. Effect sizes for the Mann–Whitney *U* test results were calculated using the formula *r* = z/√n, where ‘*z*’ is the *z*‐statistic and ‘*n*’ is the total sample size; effect sizes were interpreted as small (*r* ≤ 0.20), medium (0.21–0.79), or large (*r* ≥ 0.80). A two‐tailed *p*‐value < 0.05 was considered statistically significant for all analyses. Statistical analyses were performed using IBM SPSS Statistics for Windows, Version 28.0 (Armonk, NY: IBM Corp) [9, 10].

## 3 Results

### 3.1 Study Population Characteristics

**FIGURE 2:**
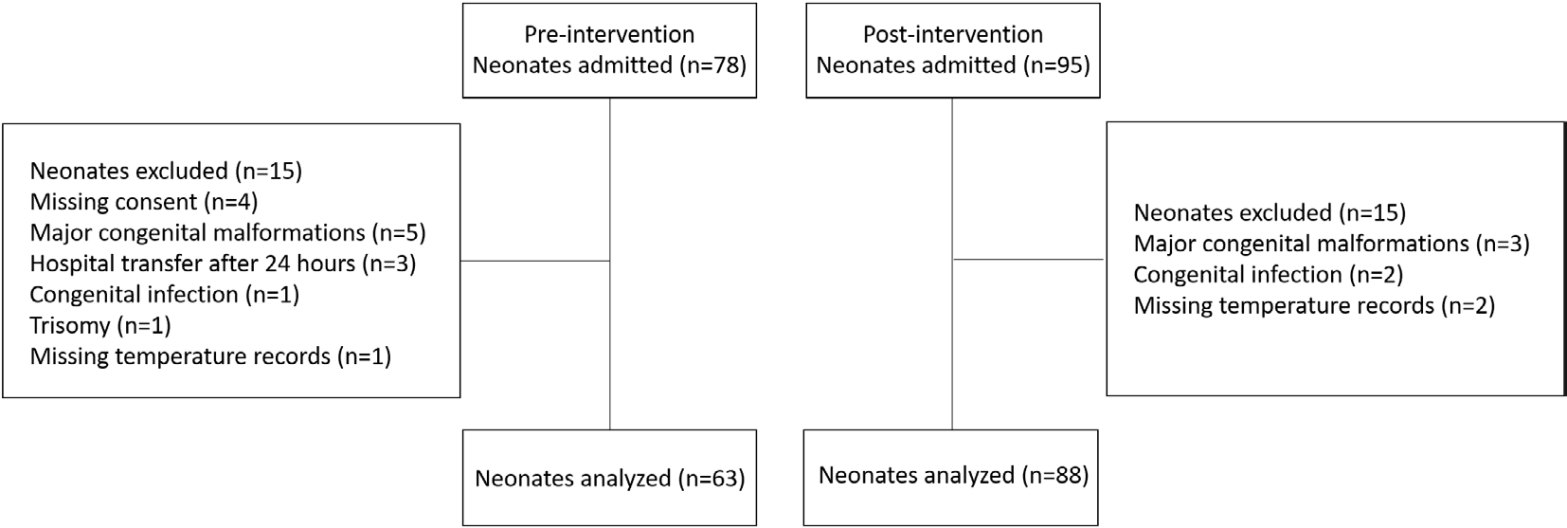
Flowchart of participant inclusion and exclusion in the study.

During the pre‐intervention period (July–December 2021), 78 neonates met the initial eligibility criteria. Of these, 15 were excluded due to various reasons: lack of parental consent (*n* = 4), presence of major congenital malformations (*n* = 5), transfer from another hospital after 24 h of life (*n* = 3), congenital infection (*n* = 1), confirmed trisomy (*n* = 1), and missing initial temperature records (*n* = 1). In the post‐intervention period (January–June 2023), 95 neonates were initially eligible, with 7 subsequently excluded due to congenital infections (*n* = 2), major congenital malformations (*n* = 3), or missing initial temperature records (*n* = 2). Consequently, the final analysis included 151 preterm neonates: 63 in the pre‐intervention group and 88 in the post‐intervention group (Figure 2).

### 3.2 Comparison of Maternal and Neonatal Baseline Characteristics

Table 1 details the baseline maternal and neonatal characteristics for both study periods. The only statistically significant difference between the groups was a higher proportion of mothers receiving antenatal corticosteroids in the post‐intervention period (81.8%) compared to the pre‐intervention period (65.1%) (*χ* ^2^ = 5.64, *p* = 0.018; PR = 1.26; 95% CI: 1.03–1.55). Otherwise, the groups were similar in all other measured maternal and neonatal characteristics (Table 1).

**TABLE 1:** Characteristics of pregnant women and neonates participating in the study before and after the intervention (*n* = 151).

Variables	Pre‐intervention	Post‐intervention	*p* ^a^
Qualitative variables	*n* (%)	*n* (%)	
Maternal age	63 (100.0)	88 (100.0)	0.305
< 20 years	8 (12.7)	14 (15.9)	
20–35 years	46 (73.0)	54 (61.4)	
> 35 years	9 (14.3)	20 (22.7)	
Hypertensive syndromes in pregnancy	63 (100.0)	88 (100.0)	0.728
Yes	29 (46.0)	38 (43.2)	
No	34 (54.0)	50 (56.8)	
Gestational diabetes	63 (100.0)	88 (100.0)	0.656
Yes	11 (17.5)	13 (14.8)	
No	52 (82.5)	75 (85.2)	
Urinary tract Infection	63 (100.0)	88 (100.0)	0.585
Yes	16 (25.4)	19 (21.6)	
No	47 (74.6)	69 (78.4)	
Antenatal Corticosteroid	63 (100.0)	88 (100.0)	**0.019**
No	22 (34.9)	16 (18.2)	
1 or 2 doses	41 (65.1)	72 (81.8)	
Neonate sex	63 (100.0)	88 (100.0)	0.967
Male	32 (50.8)	45 (51.1)	
Female	31 (49.2)	43 (48.9)	
Gestational age at birth	63 (100.0)	88 (100.0)	0.126
Up to 28 weeks and 6 days	30 (47.6)	31 (35.2)	
29 weeks or more	33 (52.4)	57 (64.8)	
Birth weight	63 (100.0)	88 (100.0)	0.232
Up to 999 g	26 (41.3)	28 (31.8)	
1.000 g or more	37 (58.7)	60 (68.2)	
Small for gestational age	63 (100.0)	87 (100.0)	0.636
Yes	18 (28.6)	28 (32.2)	
No	45 (71.4)	59 (67.8)	
Type of delivery	63 (100.0)	88 (100.0)	0.278
Cesarean	42 (66.7)	51 (58.0)	
Vaginal	21 (33.3)	37 (42.0)	
Type of pregnancy	63 (100.0)	88 (100.0)	0.663
Multiple	14 (22.2)	17 (19.3)	
Singleton	49 (77.8)	71 (80.7)	
CPAP in delivery room	63 (100.0)	88 (100.0)	0.649
Yes	32 (50.8)	48 (54.5)	
No	31 (49.2)	40 (45.5)	
Resuscitation in delivery room	63 (100.0)	88 (100.0)	0.196
Yes	32 (50.8)	54 (61.4)	
No	31 (49.2)	34 (38.6)	
Apgar score at first minute	61 (100.0)	87 (100.0)	0.118
Less than 7 points	25 (41.0)	47 (54.0)	
7 points or more	36 (59.0)	40 (46.0)	
Apgar Score at fifth minute	61 (100.0)	88 (100.0)	0.173
Less than 7 points	9 (14.8)	21 (23.9)	
7 points or more	52 (85.2)	67 (76.1)	

Quantitative variables	Median (IQR)		*p* ^a^
Maternal age. years	29 (13)	27 (12)	0.420
Prenatal visits. number	5 (2)	5 (2)	0.687
Gestational age at birth. weeks	29.3 (5.4)	30.5 (4.9)	0.326
Birth weight. grams	1.182.5 (672.5)	1.175 (756.3)	0.839
Birth weight *z*‐score	−0.06 (1.97)	−0.48 (1.73)	0.108

*Note:* (1) Categorical data are expressed as absolute (*n*) and relative (%) frequencies; (2) Continuous data are expressed as median and interquartile range. Bold values indicate significance at *p* < 0.05.

Abbreviations: g, grams; IQR, interquartile range; *n*, number; qtd, quantity.

^a^ The significance of differences between groups was determined using the Chi‐square test or Mann–Whitney *U* test.

### 3.3 Impact on Hypothermia Prevalence

The primary outcome, prevalence of admission hypothermia (< 36.5°C), was significantly lower in the post‐intervention period (68.2%, 95% CI: 57.9%–77.3%) compared to the pre‐intervention period (82.5%, 95% CI: 71.7%–90.5%). This represents a statistically significant reduction (*χ* ^2^ = 3.95, *p* = 0.047; PR = 0.83, 95% CI: 0.69–0.99) (Table 2). Among the neonates who experienced hypothermia, there were no significant differences between the pre‐ and post‐intervention groups regarding median gestational age (*p* = 0.377), median birth weight (*p* = 0.633), or the distribution of hypothermia severity (mild vs. moderate) (*p* = 0.903). Notably, no instances of severe hypothermia (< 32.0°C) were recorded in either period (Table 2).

**TABLE 2:** Comparison of hypothermia and related variables before and after intervention (*n* = 151).

Variable	Pre‐intervention		Post‐intervention		*p* ^a^
	*n* (%)	Prevalence (95% CI)	*n* (%)	Prevalence (95% CI)	
Hypothermia	63 (100.0)		88 (100.0)		**0.047**
Yes	52 (82.5)	82.5% (71.7%–90.5%)	60 (68.2)	68.2% (57.9%–77.3%)	
No	11 (17.5)		28 (31.8)		
Gestational age	52 (100.0)		60 (100.0)		0.377
Up to 28 weeks and 6 days	26 (50.0)	50.0% (36.6%–63.4%)	25 (41.7)	41.7% (29.7%–54.4%)	
29 weeks or more	26 (50.0)		35 (58.3)		
Birth weight	52 (100.0)		60 (100.0)		0.633
Up to 999 g	24 (46.2)	46.2% (33.0%–59.7%)	25 (41.7)	41.7% (29.7%–54.4%)	
1.000 g or more	28 (53.8)		35 (58.3)		
Degree of hypothermia	52 (100.0)		60 (100.0)		0.903
Moderate (32°C to 35.9°C)	42 (80.8)	80.8% (68.4%–89.8%)	49 (81.7)	81.7% (70.4%–89.9%)	
Mild (36°C to 36.4°C)	10 (19.2)		11 (18.3)		

*Note:* (1) Categorical data are expressed as absolute (*n*) and relative (%) frequencies; (2) For gestational age, birth weight, and degree of hypothermia variables, calculations were performed considering only neonates who presented with hypothermia. Bold values indicate significance at *p* < 0.05.

Abbreviations: CI, confidence interval; g, grams; *n*, number.

^a^ The significance of differences between groups was determined using the Chi‐square test (hypothermia/gestational age/birth weight/degree of hypothermia vs. study period).

### 3.4 Impact on Admission Temperature

**FIGURE 3:**
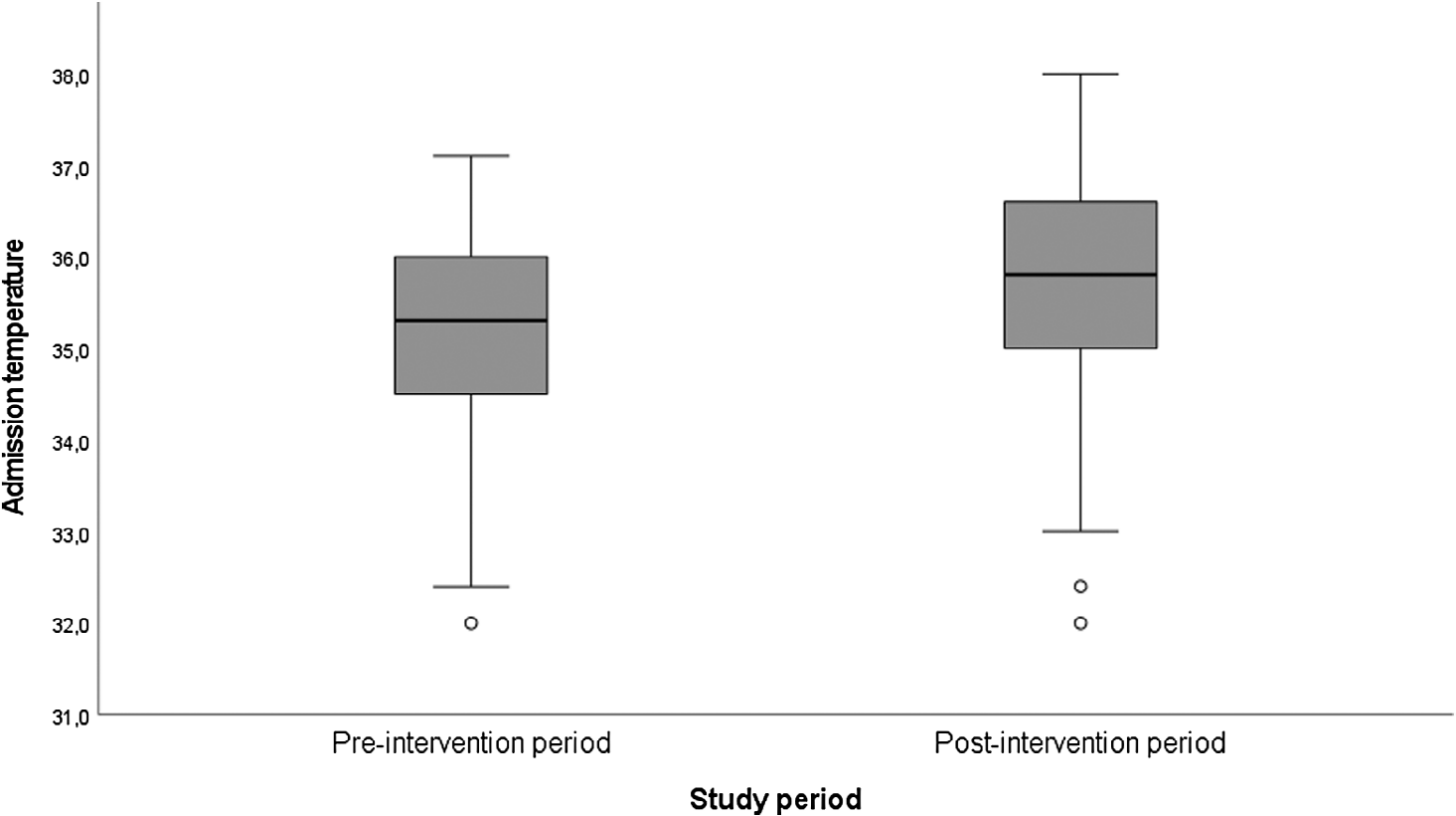
Admission temperature before and after the intervention project (*n* = 151).

Neonates admitted during the post‐intervention period exhibited significantly higher median admission temperatures (35.8°C, IQR = 1.6°C) compared to those admitted during the pre‐intervention period (35.3°C, IQR = 1.5°C). This difference was statistically significant (Mann–Whitney *U* = 2113, *z* = −2.488, *p* = 0.013), although the calculated effect size was small (*r* = 0.20) (Figure 3).

### 3.5 Association Between Hypothermia and Mortality

In the post‐intervention group, there was a statistically significant association between admission hypothermia and neonatal mortality (*χ* ^2^ = 5.677, *p* = 0.017). Among these neonates, mortality was significantly higher in those who were hypothermic (35.0%) compared to those who were normothermic (10.7%) (PR = 3.27; 95% CI: 1.06–10.05) (Table 3). This association was primarily driven by deaths occurring in the early neonatal period (days 0–6).

**TABLE 3:** Assessment of the association of hypothermia in the first hour of life before and after intervention and the outcome of death (*n* = 151).

Variables	Hypothermia pre‐intervention			Hypothermia post‐intervention		
	Yes	No	*p* ^a^	Yes	No	*p* ^a^
Outcome			0.401			**0.017**
Death	16 (30.8)	2 (18.2)		21 (35.0)	3 (10.7)	
Hospital discharge	36 (69.2)	9 (81.8)		39 (65.0)	25 (89.3)	
Mortality			—*			—*
Early neonatal	9 (56.3)	1 (50.0)		14 (66.7)	1 (33.3)	
Late neonatal	3 (18.8)	0 (0.0)		6 (28.6)	2 (66.7)	
Post‐neonatal	4 (25.0)	1 (50.0)		1 (4.8)	0 (0.0)	

*Note:* Qualitative data are expressed as absolute (*n*) and relative (%) frequencies. Bold values indicate significance at *p* < 0.05. Prerequisites for applying the statistical test were not met. Some variables had missing values.

^*^ The symbol asterisk indicates that the prerequisites for applying the statistical test were not met.

^a^ The significance of differences between groups was determined using the Chi‐square test (qualitative variables).

## 4 Discussion

This quasi‐experimental study found that the implementation of a targeted QI intervention was associated with a significant reduction in the prevalence of admission hypothermia among vulnerable preterm neonates. Furthermore, the intervention led to a statistically significant, albeit modest, increase in median admission temperatures. Notably, a significant association between admission hypothermia and early neonatal mortality was observed following the intervention period, highlighting the clinical relevance of thermal stability in this population.

The observed decrease in hypothermia prevalence following the QI initiative is consistent with findings from other studies in resource‐constrained settings. Quality improvement strategies that use standardized protocols and focused multidisciplinary training have consistently been effective in improving thermal care [11, 12].

However, the residual hypothermia prevalence in our cohort remained considerably higher than rates reported in comparable neonatal units in Rwanda [3] and Ethiopia [4], suggesting that persistent challenges or barriers may have attenuated the full potential impact of the intervention within our specific context. Importantly, baseline risk factors for hypothermia, such as prematurity, low birth weight, and the need for delivery room resuscitation, which are well‐documented contributors [2, 3], were similarly distributed between the study periods, suggesting that the observed improvement may be attributable to the intervention itself rather than changes in patient characteristics.

While the increase in median admission temperature was statistically significant, the magnitude of this improvement was relatively small. Research from multicenter cohorts, particularly in low‐ and middle‐income countries, suggests that more substantial increases in admission temperature are needed to achieve clinically meaningful reductions in mortality [13]. The emergence of a significant association between hypothermia and early neonatal mortality only in the post‐intervention period warrants careful consideration. This finding might reflect improved detection or recording of outcomes following heightened staff awareness due to the QI training, rather than a true increase in risk. Nevertheless, the established link between hypothermia and severe adverse outcomes like sepsis and intraventricular hemorrhage [2] reinforces the critical importance of achieving normothermia as a fundamental indicator of quality neonatal care.

The QI intervention implemented at NICU, which combined a formal SOP with staff education through workshops and simulation, may have contributed to improved adherence to evidence‐based thermoregulation practices, such as appropriate use of occlusive wrapping and better control of the thermal environment [6]. Similar care bundle approaches have demonstrated success in reducing hypothermia rates in preterm infants [14].

Despite these efforts, the persistence of a high hypothermia rate post‐intervention suggests remaining obstacles. These may include inconsistencies in protocol adherence across all staff or shifts, as well as infrastructural limitations, such as equipment availability or maintenance, factors also identified as barriers in other resource‐limited settings [15]. Unlike high‐income countries where advanced resources generally lead to lower hypothermia prevalence, middle‐income settings like Brazil often grapple with systemic challenges, including resource constraints and staff turnover, which likely influenced the overall impact achievable by our intervention.

Several limitations should be acknowledged when interpreting these findings. The single‐center design inherently limits the generalizability of the results to other NICU with different patient populations, resources, or practice patterns. The relatively small sample size constrained statistical power, particularly for subgroup analyses, such as examining mortality by specific cause or severity of hypothermia. The retrospective nature of data collection introduces potential for information bias, especially concerning the exclusion of infants with missing temperature data. Furthermore, the quasi‐experimental pre‐post design, lacking a concurrent control group, cannot definitively rule out the influence of unmeasured confounding factors or secular trends occurring between the two study periods.

In addition, although delivery‐room ambient temperature targets were defined in the SOP and routinely monitored during clinical care, the absence of systematic quantitative recording of achieved room temperatures precluded formal analysis of adherence to environmental temperature targets. Consequently, the observed improvements in neonatal thermoregulation should be attributed to the combined implementation of the bundle components rather than any single environmental factor. Finally, the evaluation period following the intervention was relatively short, precluding assessment of the long‐term sustainability of the observed improvements.

Despite these limitations, this study provides valuable insights into the effectiveness of a structured QI approach to neonatal thermoregulation in a resource‐limited tertiary NICU. The findings underscore the practical implication that standardized protocols coupled with ongoing staff education are essential components for reducing neonatal hypothermia. The observed link between residual hypothermia and mortality reinforces the urgency of optimizing thermal care to improve infant survival. Future research should prioritize multicenter, controlled study designs with extended follow‐up periods to confirm these findings, assess the intervention's durability, and evaluate its impact on other critical neonatal morbidities, such as sepsis and neurodevelopmental outcomes [2].

In practice, sustained improvement will require ongoing staff training, dedicated resources for essential equipment and infrastructure, and regular audits of protocol adherence to ensure consistent application of best practices and to move closer to the outcomes achieved in higher‐resource settings [16].

## 5 Conclusion

This study demonstrated that the implementation of a targeted QI intervention was effective in significantly reducing the prevalence of admission hypothermia and modestly increasing admission temperatures among preterm neonates in a resource‐limited NICU setting.

Despite these improvements, the persistence of high hypothermia rates underscores that further sustained efforts, including enhanced staff training, infrastructural support, and potentially broader systemic changes, are necessary. Future research employing robust, multicenter designs is warranted to confirm these findings, evaluate long‐term impacts on neonatal morbidity and mortality, and identify strategies to overcome implementation barriers in similar contexts.

## Author Contributions

P.M.G.F.R.: Conceptualization, methodology, formal analysis, writing – original draft. J.D.A.S.C.: Conceptualization, software, data curation, formal analysis, investigation, writing – original draft. S.F.C.: Conceptualization, methodology, software, data curation, investigation, writing – review and editing. A.T.B.S.: Investigation, resources, supervision, writing – review and editing. C.A.F.: Data curation, software, data curation, formal analysis, writing – review and editing. A.K.C.B.: Resources, supervision, writing – review and editing. R.C.A.S.: Validation, writing – review and editing. C.L.F.: Investigation, supervision, writing – review and editing. R.N.C.: Methodology, supervision, writing – review and editing. F.A.F.: Conceptualization, supervision, writing – review and editing. A.C.N.G.B.: Conceptualization, supervision, funding acquisition, writing – review and editing.

## Funding

This work was supported by Coordenação de Aperfeiçoamento de Pessoal de Nível Superior.

## Disclosure

The authors have nothing to report.

## Ethics Statement

This study received ethical approval from the Research Ethics Committee of the Hospital (CAAE registration number: 11315419.6.0000.5292). Written informed consent was obtained from the parents or legal guardians of all participating neonates prior to data collection. All patient data were fully anonymized to ensure confidentiality. The study procedures adhered strictly to the ethical principles outlined in Brazilian National Health Council Resolution No. 466/2012 and complied with the requirements of the Brazilian General Data Protection Law (Law No. 13.709/2018).

## Conflicts of Interest

The authors declare no conflicts of interest.

## Supporting information


**Data S1:** Quality improvement protocol.

**Data S2:** STROBE statement—checklist of items that should be included in reports of observational studies.

## Data Availability

The data that support the findings of this study are available from the corresponding author upon reasonable request.
