# Supplementary material for: Quality Improvement Intervention Lowers Hypothermia Rates in Preterm Infants in a Resource‐Limited Setting
Source: Pediatr Int. 2026 Apr 15;68(1):e70397. doi: 10.1111/ped.70397 (PMC13081098; doi:10.1111/ped.70397)
Supplement: Supplementary file 1 — Data S1: Quality improvement protocol. [file PED-68-e70397-s002.docx]

**Supplementary Material.** Quality Improvement Protocol.

**Title**: Prevention of Neonatal Hypothermia in Delivery Care Settings
**Version**: 1
**Issue Date**: 07 January, 2023
**Next Review**: 07 January, 2025

**1. OBJECTIVES**

- Adopt best practices for maintaining newborn temperature post-birth.
- Prevent thermal dysregulation and its complications in the Obstetric Center (OC) and Obstetric Surgical Center (OSC).
- Describe general care practices for healthcare teams to prevent and/or address thermal dysregulation in newborns in delivery care settings.
- Establish strategies to achieve normothermia in newborns in the OC and OSC.

**2. MATERIALS**

- 1 disposable face mask
- 1 digital thermometer
- 1 digital room thermometer
- 1 skin sensor cable compatible with incubator and Radiant Heat Cradle (RHC)
- Water for hand washing
- Soap for hand hygiene
- Paper towels
- 1 multiparameter monitor temperature sensor (in the absence of an incubator/RHC skin sensor)
- 1 bottle of 1000 mL double-distilled water
- 1 cotton cap (small, medium, or large)
- 1 polyethylene bag (30 x 50 cm)
- 1 cloth cap (wool or cotton)
- 1 plastic cap
- 1 pair of non-sterile procedure gloves
- 1 pair of sterile procedure gloves
- Cotton, preferably orthopedic
- 1 crepe bandage
- 1 pack of gauze
- Plastic wrap
- Hypoallergenic porous tape
- 1 transparent dressing
- 1 incubator or RHC
- 1 neonatal thermal mattress or blanket

**3. PROCEDURE DESCRIPTION**

**3.1 General Information**

Maintaining and controlling the body temperature of newborns, especially preterm infants, is critical for their survival. These infants are prone to rapid cooling or overheating, as their body temperature passively follows environmental changes.

The Brazilian Society of Pediatrics (SBP) recommends maintaining delivery room temperatures above 23°C, while the World Health Organization (WHO) advises 26°C during neonatal resuscitation.

- **Normothermia**: 36.5°C to 37.5°C
  The WHO recommends that stable newborns be placed in skin-to-skin contact with their mothers during the first hour after birth (the "golden hour") to prevent hypothermia and promote breastfeeding. Skin-to-skin contact and breastfeeding should be initiated as soon as possible.

The WHO defines newborn temperature ranges and classifies hypothermia by severity:

- **Mild Hypothermia (Potential Cold Stress)**: 36.0°C to 36.4°C
- **Moderate Hypothermia**: 32.0°C to 35.9°C
- **Severe Hypothermia**: Below 32.0°C
- **Hyperthermia**: Above 37.5°C

**3.2 Task and Care Description in the Obstetric Surgical Center and Obstetric Center**

- Maintain the ambient temperature of the surgical and delivery rooms above 23°C.
- Keep doors closed and minimize personnel movement to reduce air currents.
- Measure maternal temperature every 2 hours during active labor and the expulsion phase in the OC, and before anesthesia in the OSC. If this is not feasible, measure at least once during active labor.
- Ensure maternal thermal comfort during labor and surgical preparation through the use of blankets, maintaining ambient temperatures above 23°C, and other comfort measures.
- Preheat the radiant heat source for at least 30 minutes before birth and pre-warm the surgical fields.
- Receive the newborn on pre-warmed fields and place under a radiant heat source.
- Use a cloth cap (wool or cotton) for term infants to reduce heat loss through the fontanelle.
- For preterm infants, use a double cap: cover the scalp with plastic first, then place a wool or cotton cap over it.
- Inform the mother about the importance of the golden hour and obtain her consent.
- Ensure the Golden Hour: Place stable newborns in skin-to-skin contact on the mother’s chest, covered with a warmed cloth, during the first hour after birth (see specific Golden Hour protocol).
- Support breastfeeding initiation as soon as the newborn shows readiness, if desired by the mother.
- For newborns requiring pediatric care at birth, provide skin-to-skin contact with the mother for 1 hour once they are clinically stable.
- Defer administration of vitamin K, PVP-I eye drops, and anthropometric measurements (weight, length, head circumference, abdominal circumference, and chest circumference) until after the Golden Hour.
- Weigh the newborn wrapped in a pre-weighed, warmed sheet to account for the sheet’s weight (see specific protocol).
- Delay bathing until at least 24 hours after birth, unless otherwise indicated by medical recommendation.
- For clinically stable preterm infants under 34 weeks, place in skin-to-skin contact with the mother under continuous supervision by a pediatrician or nurse.
- For clinically unstable preterm infants under 34 weeks or weighing less than 1,500 g at birth: do not dry the infant; place them in a polyethylene bag, which should remain in place until thermal stabilization in the neonatal unit.
- Provide heated respiratory gases when necessary.
- Use a chemical thermal mattress, if available, for preterm infants with an estimated weight below 1,000 g.
- Maintain the axillary temperature of preterm infants between 36.5°C and 37.5°C (normothermia) from birth to admission to the neonatal unit.
- Follow intra- and inter-hospital transport protocols (see specific protocol), including:
  - For preterm infants under 34 weeks, transfer from the OC/OSC to the neonatal unit in a double-walled transport incubator, kept charged and connected to the power supply with a programmed temperature of 35°C to 37°C until transport.
  - Keep the plastic bag covering the infant’s body and the double cap (plastic and cotton/wool) in place during transport.

**4. REFERENCES**

- ALMEIDA, M.F.B.; GUINSBURG, R. *Neonatal Resuscitation <34 Weeks in the Delivery Room: 2016 Guidelines of the Brazilian Society of Pediatrics*. 2016.
- ALMEIDA, M.F.B.; GUINSBURG, R. *Neonatal Resuscitation ≥34 Weeks in the Delivery Room: 2016 Guidelines of the Brazilian Society of Pediatrics*. 2016.
- BRAZIL. Ministry of Health. *Newborn Healthcare: Guide for Healthcare Professionals*. 2nd ed. Brasília: Ministry of Health, 2012. Vol. 1. Available at: https://bvsms.saude.gov.br/bvs/publicacoes/atencao_saude_recem_nascido_profissionais_v1.pdf.
- BRAZIL. Ministry of Health. *Newborn Healthcare: Guide for Healthcare Professionals*. 2nd ed. Brasília: Ministry of Health, 2014. Vol. 4. Available at: http://bvsms.saude.gov.br/bvs/publicacoes/atencao_saude_recem_nascido_v4.pdf.
- BRAZIL. Ministry of Health. *Humanized Newborn Care: Kangaroo Method: Technical Manual*. 3rd ed. Brasília: Ministry of Health, 2017.
- BRAZIL. Ministry of Health. *Baby-Friendly Hospital Initiative*. Brasília: Ministry of Health, 2017. Available at: https://www.saude.gov.br/artigos/41186-iniciativa-hospital-amigo-da-crianca-ihac.
- CLOHERTY, J.; EICHENWALD, E.; STARK, A. *Manual of Neonatology*. 7th ed. Rio de Janeiro: Guanabara Koogan; 2015.
- COSTA, C.C.; TONETE, V.L.P.; PARADA, C.M.G.L. Knowledge and practices in handling neonatal incubators by nursing professionals. *Acta Paulista de Enfermagem*. 2017; 30(2):174-80. Available at: http://www.scielo.br/pdf/ape/v30n2/1982-0194-ape-30-02-0174.pdf.
- DARMSTADT, G.L.; BHUTTA, Z.A.; COUSENS, S.; ADAM, T.; WALKER, N.; BERNIS, L. Evidence-based, cost-effective interventions: how many newborn babies can we save? *The Lancet*. 2005; 365:977-88. Available at: https://www.ncbi.nlm.nih.gov/pubmed/15767001.
- *Manual of Breastfeeding Standards and Routines of HU-UFGD/EBSERH*. 2017. 102 pages. Approved by Ordinance 22 on February 22, 2019, published in Service Bulletin No. 178, February 25, 2019, annexed to Ordinance No. 22. Available at: http://www.ebserh.gov.br/web/hu-ufgd/superintendencia/ccne/comissoes/comissao-de-incentivo-e-apoio-ao-aleitamento-materno-ciaam.
- WORLD HEALTH ORGANIZATION. *WHO Safe Childbirth Checklist Implementation Guide: Improving the Quality of Facility-Based Delivery for Mothers and Newborns*. Geneva, 2017.
- WORLD HEALTH ORGANIZATION. *WHO Recommendations: Intrapartum Care for a Positive Childbirth Experience*. Geneva: World Health Organization; 2018. License: CC BY-NC-SA 3.0 IGO. Translated by Miriam Rêgo de Castro – Obstetric Nurse, Bom Parto Team.
- RIBEIRO, M.A.C.R. *Undergraduate Nursing Thesis, JK College, Taguatinga-DF, 2005. Aspects Influencing Thermoregulation: Nursing Care for Preterm Newborns*.
- WORLD HEALTH ORGANIZATION. *WHO Recommendations for Care of the Preterm or Low-Birth-Weight Infant*. Geneva: World Health Organization; 2018. License: CC BY-NC-SA 3.0 IGO. Translated by Miriam Rêgo de Castro – Obstetric Nurse, Bom Parto Team.
